# Shear bond strength analysis of self-etch and total-etch adhesive systems on antioxidant-treated dentin in post-endodontic restoration

**DOI:** 10.3389/fdmed.2026.1809570

**Published:** 2026-04-08

**Authors:** Suvarna Kavya Harish, Shruthi H. Attavar

**Affiliations:** Nitte (Deemed to be University), AB Shetty Memorial Institute of Dental Sciences (ABSMIDS), Department of Conservative Dentistry and Endodontics, Mangalore, India

**Keywords:** antioxidants, dentin-bonding agents, post-endodontic restoration, self-etch adhesive, sodium hypochlorite, total-etch adhesive

## Abstract

**Aim:**

To evaluate the effect of antioxidant pretreatment on the shear bond strength and failure modes of total-etch and self-etch adhesive systems applied to sodium hypochlorite (NaOCl)–treated dentin.

**Materials and methods:**

Eighty extracted human premolars were prepared and irrigated with NaOCl. The specimens were allocated into two groups based on the adhesive strategy employed: total-etch and self-etch. Each group was further subdivided according to antioxidant pretreatment: control (no antioxidant), sodium ascorbate, alpha-tocopherol, and nanocurcumin. Composite resin was bonded, and shear bond strength was tested using a universal testing machine. Failure modes were evaluated microscopically. Data were analyzed using one-way ANOVA with *post-hoc* tests, two-way ANOVA to assess the effects of antioxidant type and adhesive system, and chi-square analysis for failure modes *(P* *<* *0.05).*

**Results:**

NaOCl-treated dentin without antioxidant pretreatment showed the lowest bond strength values in both adhesive systems. Sodium ascorbate and nanocurcumin significantly improved bond strength compared with control and alpha-tocopherol, with no significant difference between them. Self-etch adhesives demonstrated lower baseline bond strength but greater recovery following antioxidant pretreatment. Two-way ANOVA revealed significant effects of antioxidant type, adhesive system, and their interaction. Control groups predominantly exhibited adhesive failures, while antioxidant-treated groups showed increased mixed and cohesive failures.

**Conclusion:**

Antioxidant pretreatment effectively restores bonding to NaOCl-treated dentin. Sodium ascorbate and nanocurcumin demonstrated comparable efficacy, with greater benefit observed for self-etch adhesives.

## 1 Introduction

Tooth decay in advanced stages leads to extensive loss of coronal tooth structure, often necessitating endodontic intervention to preserve tooth function (1). Endodontically treated teeth commonly exhibit compromised structural integrity due to the cumulative effects of caries, trauma, removal of infected dentin, and access cavity preparation and canal instrumentation, resulting in altered biomechanical properties, increased fracture susceptibility, and compromised aesthetics (2). Consequently, achieving an effective coronal restoration after root canal therapy is essential for long-term clinical success (3, 4). The primary objectives of post-endodontic restoration include providing an adequate coronal seal, restoring mechanical strength and occlusal function, improving aesthetics, and minimizing the risk of fracture, as coronal microleakage remains a major cause of endodontic failure (3, 5).

Resin composite is widely used for restoring endodontically treated teeth when sufficient tooth structure remains because of its favorable aesthetics, conservative preparation, and micromechanical bonding to enamel and dentin (6). It is also commonly used as an interim restoration before full-coverage restorations (3). The success of composite restorations largely depends on the performance of the adhesive system and the condition of the dentin substrate (7). Adhesive systems are classified as total-etch or self-etch based on their interaction with the smear layer and dentin (8). Total-etch systems remove the smear layer using phosphoric acid, whereas self-etch systems utilize acidic monomers that concurrently demineralize and infiltrate dentin, influencing hybrid layer formation and bond durability, particularly on chemically altered substrates (1, 7).

During endodontic treatment, sodium hypochlorite (NaOCl) is commonly employed during endodontic procedures because of its potent antimicrobial properties and its capacity to dissolve organic tissue (9). However, NaOCl adversely affects subsequent adhesive bonding by generating residual oxygen free radicals that interfere with free-radical polymerization of adhesive monomers, resulting in compromised hybrid layer formation, reduced micromechanical retention, and significantly lower bond strength on NaOCl-treated dentin (10–12).

To reverse these effects, antioxidant pretreatment of dentin has been proposed to neutralize residual oxidizing agents and restore the redox potential of the substrate (11). Sodium ascorbate, the most extensively investigated antioxidant, has been shown to significantly improve bond strength of both total-etch and self-etch adhesive systems following NaOCl exposure by facilitating normal resin polymerization and hybrid layer formation (1, 3, 10, 13). Other antioxidants, including alpha-tocopherol, have also demonstrated improvements in bond strength of chemically altered dentin, suggesting their potential as dentin pretreatment agents (14, 15).

Curcumin, a natural polyphenol derived from Curcuma longa, exhibits antioxidant and antimicrobial properties; however, its clinical application is limited by poor aqueous solubility and low bioavailability (16). Nanocurcumin formulations overcome these limitations by enhancing solubility and biological activity, though their role in adhesive dentistry remains largely unexplored (16, 17).

While sodium ascorbate and alpha-tocopherol have been widely studied for improving bonding to NaOCl-treated dentin, limited evidence exists regarding the effect of nanocurcumin, particularly across different adhesive strategies. Given the mechanistic differences between total-etch and self-etch systems, antioxidant efficacy may vary with the adhesive approach. Therefore, the present study aimed to evaluate and compare the shear bond strength and failure modes of self-etch and total-etch adhesives applied to NaOCl-treated dentin following pretreatment with sodium ascorbate, alpha-tocopherol, and nanocurcumin.

## 2 Material and methods

### 2.1 Protocol and registration

This study obtained the institutional ethical clearance (Cert. No: ETHICS/ABSMIDS/448/2024).

The sample size was calculated using nMaster software (Version 2.0) with 80% power and 5% level of significance. Based on the effect size derived from previous studies, a minimum of 160 specimens (*n* = 20 per subgroup) was required.

A total of 80 extracted non-carious human maxillary and mandibular premolars were collected from the Department of Oral and Maxillofacial Surgery, AB Shetty Memorial Institute of Dental Sciences. Teeth were cleaned, disinfected and handled as per the recommendations and guidelines laid down by Occupational safety and Health Administration (OSHA) and Center for Disease Control and prevention and were stored in closed bottle in saline.

Teeth were sectioned longitudinally to obtain two dentin specimens from each tooth (total *n* = 160 specimens). To control for inter-tooth variability, one half from each tooth was allocated to the total-etch group and the corresponding half to the self-etch group, ensuring that both adhesive systems were tested on dentin derived from the same tooth.

Within each adhesive group, specimens were further subdivided according to antioxidant pretreatment (*n* = 20 per subgroup). The sectioned specimens were considered the experimental units for statistical analysis.

### 2.2 Sample preparation

Each tooth was vertically sectioned in the mesiodistal direction through the pulp chamber into two, using a diamond disk (Mani, Tochigi, Japan) and low speed handpiece (NSK, Tochigi, Japan) under copious water spray.

The sections were mounted in an acrylic block with the root submerged in acrylic.

All the sectioned samples were immersed in a solution of 3% NaOCl for 2 min, followed by rinsing with distilled water.

### 2.3 Formulation of antioxidants

#### 2.3.1 Sodium ascorbate (SA)

To prepare the 10% SA solution, 10 g of SA (Cynor Laboratories, Gujarat, India) was dissolved in 100 mL of distilled water to make 10% SA solution (10).

#### 2.3.2 Alpha-tocopherol

To prepare 20% alpha-tocopherol solution, 20 g of alpha-tocopherol powder (DI-a-Tocopherol acetate RESEARCH-LAB FINE CHEM INDUSTRIES, Mumbai, India) was mixed with 100 mL of ethyl alcohol (15).

#### 2.3.3 Nanocurcumin

To prepare nanocurcumin, 600 mg of nanocurcumin powder was weighed and dissolved in 10 mL of distilled water to prepare a solution with concentration 60 mg/mL (Ramini Bio Nutrition Pvt. Ltd., Hyderabad, India) (16).

The concentrations were selected based on previously published studies demonstrating antioxidant efficacy in dental applications.

### 2.4 Sample allocation

The specimens randomly allocated into two main experimental groups based on the adhesive strategy employed:

Group A: Total-etch adhesive system

Group B: Self-etch adhesive system

Each primary group was subsequently subdivided into four distinct subgroups. (*n* = 20 per subgroup) according to the dentin surface pretreatment protocol:

Subgroup 1: Control (NaOCl treatment without antioxidant application)

Subgroup 2: Sodium ascorbate pretreatment

Subgroup 3: Alpha-tocopherol pretreatment

Subgroup 4: Nanocurcumin pretreatment

This resulted in a total of eight experimental subgroups.

### 2.5 Antioxidant application protocol

In Subgroups 2–4, the designated antioxidant solution (sodium ascorbate, alpha-tocopherol, or nanocurcumin) was applied using a disposable syringe to completely cover the exposed dentin surface. The solution was allowed to remain in contact with the dentin for 5 min. A 5-minute application time was selected for both antioxidants to ensure adequate radical neutralization while maintaining clinical feasibility and chairside practicality. During this period, the solution was replenished as needed to ensure continuous surface coverage; however, no mechanical agitation was performed.

After 5 min, the specimens were thoroughly rinsed with distilled water for 15 s to eliminate residual antioxidant solution and gently dried using sterile cotton pellets (3, 15, 18, 19).

### 2.6 Experimental procedure

The bonding area was delimited with 3 mm diameter hole punched on an adhesive tape for the bonding procedures.

The specimens were then bonded according to their respective adhesive group.

For the self-etch groups, a one-step self-etch (7th generation) adhesive system, BeautiBond (Shofu Inc., Kyoto, Japan), with an approximate pH of 2.4, was applied to the dentin surface according to the manufacturer's instructions and light-cured for 20 s.

For the total-etch groups, the dentin surface was etched with 37% phosphoric acid for 15 s, rinsed thoroughly with distilled water, and gently air-dried to maintain moist dentin. A two-step etch-and-rinse (5th generation) adhesive system, Adper Single Bond 2 (3M ESPE, MN, USA), was then actively applied for 10 s and light-cured for 20 s.

Cylinders of composite (Beautifil II, Shofu Inc., Kyoto, Japan) at room temperature were placed on the bonded surfaces of all groups and light-cured for 40 s.

The specimens were kept in distilled water at 37 °C for 24 h before testing.

### 2.7 Sample evaluation

#### 2.7.1 Shear bond strength testing

The specimens underwent shear bond strength evaluation using a universal testing machine, with all tests performed by a single operator.

A chisel-shaped loading blade was positioned at the interface between the resin composite disc and dentin, and load was applied at a crosshead speed of 1 mm/min until bond failure occurred. The fracture load was recorded and expressed in megapascals (MPa).

#### 2.7.2 Failure mode analysis

Failure patterns were examined under a stereomicroscope at 40× magnification.

The mode of failure was categorised as adhesive (failure at the dentin–bonding agent or bonding agent–resin interface), cohesive (failure within the resin material or dentin), or mixed (involving both adhesive and cohesive components).

The frequency and percentage distribution of each failure type were calculated for all experimental groups.

### 2.8 Statistical analysis

Data were summarized using descriptive statistics and expressed as mean ± standard deviation for shear bond strength and as frequencies for failure mode distribution. Normality of the data was assessed using the Shapiro–Wilk test.

For shear bond strength- For within-group comparisons, one-way ANOVA was performed separately within the total-etch and self-etch adhesive systems to compare the effect of different antioxidants. When statistically significant differences were detected, *post-hoc* multiple comparisons were performed using Tukey's honestly significant difference (HSD) test.

For overall comparison, shear bond strength values were analyzed using two-way analysis of variance (ANOVA) to evaluate the effects of antioxidant type, adhesive system, and their interaction.

Failure mode distribution among groups was analyzed using the Chi-square test. A *P value* of < 0.05 was considered statistically significant. Statistical analysis was performed using SPSS software (version 23.0).

## 3 Results

The mean shear bond strength (SBS) values for the experimental groups are presented in Table 1. For both adhesive systems, antioxidant-treated groups demonstrated higher bond strength values than the control groups. Overall, the total-etch adhesive system exhibited higher mean bond strength values than the self-etch system.

**Table 1:** Mean ± standard deviation of shear bond strength (MPa) with one-way ANOVA results for total-etch and self-etch adhesive systems.

Adhesive system	Subgroup	Mean (MPa)	SD	*p* value
Total-etch	Control	12.70	1.86	<0.001
Total-etch	Sodium ascorbate	16.41	0.51	
Total-etch	Alpha tocopherol	14.98	0.34	
Total-etch	Nanocurcumin	17.06	0.15	
Self-etch	Control	10.45	0.41	<0.001
Self-etch	Sodium ascorbate	17.55	2.95	
Self-etch	Alpha tocopherol	14.55	0.96	
Self-etch	Nanocurcumin	16.36	1.01	

### 3.1 One-way ANOVA (within adhesive systems)

For the total-etch adhesive system, one-way ANOVA demonstrated a statistically significant difference among the antioxidant subgroups [F(3,76) = 77.279, *p* < 0.001].

Similarly, for the self-etch adhesive system, a statistically significant difference was observed among the subgroups [F(3,76) = 71.017, *p* < 0.001].

Post hoc Tukey analysis (Table 2) revealed that, within both adhesive systems, sodium ascorbate and nanocurcumin demonstrated significantly higher shear bond strength values compared with the control and alpha-tocopherol subgroups (*p* < 0.001). No statistically significant difference was observed between sodium ascorbate and nanocurcumin within either adhesive system (total-etch: *p* = 0.189; self-etch: *p* = 0.114).

**Table 2:** *Post hoc* multiple comparison analysis of shear bond strength within total-etch and self-etch adhesive systems.

Adhesive system	Comparison	Mean difference (MPa)	*p* value	F value
Total-etch	Control vs. Sodium ascorbate	−3.71	*p* *<* *0.001*	77.297
Total-etch	Control vs. Alpha tocopherol	−2.28	*p* *<* *0.001*	
Total-etch	Control vs. Nanocurcumin	−4.36	*p* *<* *0.001*	
Total-etch	Sodium ascorbate vs. Alpha tocopherol	1.43	*p* *<* *0.001*	
Total-etch	Sodium ascorbate vs. Nanocurcumin	−0.65	*p* *=* *0.201*	
Total-etch	Alpha tocopherol vs. Nanocurcumin	−2.08	*p* *<* *0.001*	
Self-etch	Control vs. Sodium ascorbate	−7.10	*p* *<* *0.001*	71.017
Self-etch	Control vs. Alpha tocopherol	−4.11	*p* *<* *0.001*	
Self-etch	Control vs. Nanocurcumin	−5.92	*p* *<* *0.001*	
Self-etch	Sodium ascorbate vs. Alpha tocopherol	2.99	*p* *<* *0.001*	
Self-etch	Sodium ascorbate vs. Nanocurcumin	1.18	*p* *=* *0.114*	
Self-etch	Alpha tocopherol vs. Nanocurcumin	−1.81	*p* *=* *0.005*	

### 3.2 Two-way ANOVA

Two-way ANOVA revealed a statistically significant main effect of antioxidant type [F(3,152) = 134.929, *p* < 0.001, partial *η*^2^ = 0.727] and adhesive system [F(1,152) = 7.021, *p* = 0.009, partial *η*^2^ = 0.044] on shear bond strength.

A significant interaction effect between antioxidant type and adhesive system was also observed [F(3,152) = 10.385, *p* < 0.001, partial *η*^2^ = 0.170], indicating that the influence of antioxidant pretreatment on bond strength differed between total-etch and self-etch systems.

The overall model explained 74.5% of the total variance in shear bond strength values (R^2^ = 0.745; adjusted R^2^ = 0.733).

### 3.3 Failure mode analysis

The distribution of failure modes is summarized in Table 3. Chi-square analysis demonstrated a statistically significant predominance of adhesive failures in the control subgroup of both adhesive systems (*p* < 0.05). In contrast, antioxidant-treated subgroups showed no statistically significant differences in failure mode distribution (*p* > 0.05), with a higher proportion of mixed failures observed.

**Table 3:** Distribution of failure modes in total-etch and self-etch adhesive systems .

Adhesive system	Subgroup	Adhesive *n* (%)	Mixed *n* (%)	Cohesive *n* (%)
Total-etch (Group 1)	Control	13 (65%)	5 (25%)	2 (10%)
	Sodium ascorbate	4 (20%)	11 (55%)	5 (25%)
	Alpha-tocopherol	7 (35%)	9 (45%)	4 (20%)
	Nanocurcumin	5 (25%)	10 (50%)	5 (25%)
Self-etch (Group 2)	Control	16 (80%)	3 (15%)	1 (5%)
	Sodium ascorbate	5 (25%)	10 (50%)	5 (25%)
	Alpha-tocopherol	9 (45%)	7 (35%)	4 (20%)
	Nanocurcumin	6 (30%)	9 (45%)	5 (25%)

## 4 Discussion

Sodium hypochlorite (NaOCl) remains the irrigant of choice in endodontic therapy because of its potent antimicrobial action and ability to dissolve organic tissues (12, 20). Despite these advantages, NaOCl has been consistently shown to adversely affect subsequent adhesive procedures by generating reactive oxygen species that interfere with free-radical polymerization of resin monomers, resulting in compromised bonding (21). In addition, NaOCl alters the chemical composition of dentin by reducing calcium and phosphate content, which weakens its mechanical properties and compromises resin-dentin micromechanical adhesion (22).

In the present study, dentin surfaces exposed to NaOCl without antioxidant pretreatment exhibited the lowest shear bond strength values (Table 1). This reduction was evident in both the adhesive systems, confirming the detrimental effect of NaOCl irrespective of the bonding strategy employed. Residual oxygen and chlorine-rich by-products inhibit polymerization of adhesive monomers, leading to incomplete resin infiltration and inadequate hybrid layer formation (11). Consequently, micromechanical retention is reduced, resulting in inferior resin-dentin adhesion when bonding is performed immediately after NaOCl irrigation without neutralization (12).

The influence of endodontic irrigants on resin-dentin bond strength has been reported inconsistently in the literature. While some investigations have reported unchanged or even enhanced bonding following NaOCl irrigation (12), most studies demonstrate a reduction in bond strength (23). These discrepancies may be attributed to differences in dentin substrate, irrigation protocols, specimen storage conditions, timing of bonding, and adhesive systems used. Importantly, the adhesive strategy itself, etch-and-rinse or self-etch, plays a critical role in determining bonding outcomes on chemically altered dentin (24).

In this study, the control subgroup of the self-etch adhesive system demonstrated significantly lower bond strength values than the corresponding total-etch control group following NaOCl exposure (Table 1). This difference was further supported by two-way ANOVA, which revealed a statistically significant main effect of adhesive system on shear bond strength *(P* *<* *0.05)*. This finding can be explained by fundamental differences in bonding mechanisms. Self-etch adhesives rely on simultaneous demineralization and resin infiltration using acidic functional monomers and incorporate the smear layer into the hybrid layer. This process is highly dependent on the integrity of the dentinal collagen matrix. The proteolytic and oxidizing effects of NaOCl degrade collagen fibrils and impair monomer diffusion, resulting in compromised hybrid layer formation and reduced bond strength.

In contrast, total-etch adhesive systems employ phosphoric acid etching to completely remove the smear layer and expose a demineralized collagen network. Although NaOCl adversely affects dentin, the aggressive etching step may partially compensate for surface alterations by increasing surface energy, dentinal permeability, and micromechanical retention. As a result, total-etch systems demonstrated comparatively better bonding performance on NaOCl-treated dentin in the absence of antioxidant pretreatment (25).

Among the antioxidant-treated groups, sodium ascorbate (subgroup 2) demonstrated significantly higher bond strength values than the control (subgroup 1) and alpha-tocopherol (subgroup 3), while showing no statistically significant difference when compared with nanocurcumin (subgroup 4) (Table 2). Two-way ANOVA revealed a highly significant main effect of antioxidant type (*P* *<* *0.001*), confirming that antioxidant efficacy was preserved irrespective of the adhesive system used. Sodium ascorbate and nanocurcumin consistently produced the greatest improvements, followed by alpha-tocopherol and the control group, and this ranking was observed in both total-etch and self-etch systems.

The superior performance of sodium ascorbate can be attributed to its strong reducing capacity, which effectively neutralizes residual oxygen free radicals generated by NaOCl, restores the redox potential of dentin, and permits normal free-radical polymerization of adhesive monomers. This facilitates effective resin infiltration and stable hybrid layer formation. The findings of the present study are consistent with previous investigations demonstrating the ability of sodium ascorbate to reverse NaOCl-induced bonding impairment (22). Celik et al. reported significant improvements across different adhesive systems, with the extent of recovery influenced by adhesive composition (3). Similarly, Dikmen et al. observed comparable improvements for both self-etch and total-etch systems following sodium ascorbate application (18).

Furuse et al. demonstrated that NaOCl-induced reductions in bond strength, particularly affecting self-etch systems, could be effectively reversed by sodium ascorbate application (26). Morris et al. further confirmed the efficacy of sodium ascorbate in reversing NaOCl-induced bonding impairment in intraradicular dentin (27).

Nanocurcumin (subgroup 4) produced bond strength values significantly higher than those of the control and alpha-tocopherol groups and comparable to sodium ascorbate. This favorable outcome may be attributed to the enhanced antioxidant capacity and improved bioavailability of curcumin when delivered in nanoparticulate form (28). Nano-encapsulation enhances solubility, stability, and diffusion, allowing effective interaction with oxidized dentin substrates (29). In addition, curcumin-based compounds have been shown to inhibit matrix metalloproteinases, preserve dentinal collagen, and improve hybrid layer stability, thereby supporting durable resin-dentin bonding (30). Although evidence regarding nanocurcumin in adhesive dentistry remains limited, the present findings suggest that it may serve as a promising alternative antioxidant for neutralizing NaOCl-induced oxidative effects.

Alpha-tocopherol (subgroup 3) demonstrated an intermediate effect, improving bond strength relative to the control group but performing significantly lower than sodium ascorbate and nanocurcumin. This comparatively reduced efficacy may be partly attributed to its hydrophobic nature, which could limit diffusion within the inherently hydrophilic dentin substrate and result in less efficient neutralization of residual oxidizing species (31). However, its performance may also have been influenced by its formulation, potential interactions with dentin surface moisture, which can affect antioxidant penetration and activity. Previous investigations have similarly reported variable or less consistent bond strength recovery with alpha-tocopherol compared with sodium ascorbate (15).

Although the magnitude of bond strength recovery differed between adhesive strategies, a consistent trend was observed across both total-etch and self-etch systems (Table 2). Sodium ascorbate and nanocurcumin consistently produced the greatest improvements, followed by alpha-tocopherol and the control group. This uniform ranking indicates that the relative efficacy of the antioxidants was preserved irrespective of adhesive type, while the extent of recovery was influenced by the bonding strategy employed, thereby reinforcing the internal validity of the study.

Following antioxidant pretreatment, self-etch adhesives demonstrated a greater recovery in bond strength compared with total-etch systems. This observation corresponds with the significant antioxidant and adhesive system interaction identified by two-way ANOVA (*P* *<* *0.05)*, indicating that the magnitude of bond strength recovery was dependent on the adhesive strategy employed. This finding suggests that self-etch adhesives are more susceptible to NaOCl-induced oxidative interference and therefore derive greater benefit from antioxidant-mediated reversal. Residual oxygen radicals disproportionately inhibit polymerization in self-etch systems due to their lower acidity and limited resin infiltration depth. Antioxidant application neutralizes these oxidizing species, restoring polymerization efficiency and improving adhesive performance. In contrast, the phosphoric acid etching step in total-etch systems may partially remove oxidized dentin, reducing but not eliminating the inhibitory effects of NaOCl, resulting in a comparatively smaller magnitude of recovery (25).

Failure mode analysis demonstrated a consistent trend across both total-etch and self-etch systems (Table 3). The control group predominantly exhibited adhesive failures, indicating a weak resin-dentin interface on NaOCl-treated dentin, whereas antioxidant-treated groups showed a shift toward mixed and cohesive failures, reflecting improved interfacial integrity. Specimens treated with sodium ascorbate and nanocurcumin exhibited the highest proportion of mixed failures, consistent with their superior bond strength values, while alpha-tocopherol showed an intermediate failure pattern. This shift in failure mode distribution supports the effectiveness of antioxidant pretreatment in restoring bonding potential and aligns with previous studies reporting reduced adhesive failures and enhanced hybrid layer stability following antioxidant application on oxidized dentin (19, 32).

Although curcumin possesses a characteristic yellow pigmentation, in the present protocol nanocurcumin was used in a controlled concentration as a transient antioxidant pretreatment and was thoroughly rinsed prior to adhesive application. Available evidence from curcumin-based photodynamic applications suggests that such formulations are not associated with clinically significant tooth discoloration (33).

Furthermore, nanoformulations demonstrate improved dispersion and reduced surface residue compared with bulk curcumin, which may decrease the likelihood of pigment retention within dentinal tubules (34). Nevertheless, the present study did not specifically evaluate discoloration potential. Future investigations are warranted to assess the effect of nanocurcumin pretreatment on dentin color and the optical properties of overlying restorations, as well as to explore whether adjunctive irrigation strategies could further minimize any possible chromatic alteration.

Within the limitations of this *in-vitro* study, antioxidants such as sodium ascorbate and nanocurcumin pretreatment following sodium hypochlorite irrigation improved immediate resin–dentin bond strength under controlled laboratory conditions, particularly for self-etch adhesive systems.

### 4.1 Clinical significance

Antioxidant pretreatment following sodium hypochlorite irrigation, particularly with sodium ascorbate or nanocurcumin, showed a positive effect on immediate resin-dentin bond strength. However, further long-term and clinical studies are required before definitive clinical recommendations can be made.

### 4.2 Limitations

Although this *in vitro* investigation provided standardized conditions to evaluate the effect of antioxidants on adhesive performance, the experimental design does not fully replicate the dynamic intraoral environment. The assessment was limited to immediate bond strength, as no aging procedures or thermocycling were performed to simulate long-term clinical conditions. Furthermore, a split-tooth design was employed, and although this reduced inter-tooth variability, complete biological independence of specimens cannot be assumed. Interfacial morphology was not evaluated using SEM or CLSM to correlate bond strength with hybrid layer characteristics, and failure mode assessment was performed by a single operator, which may introduce subjective bias.

### 4.3 Future directions

Further studies incorporating long-term aging protocols, varied antioxidant concentrations and application regimens, and *in-vivo* evaluation are warranted to better establish the clinical applicability of nanocurcumin and other antioxidant agents in adhesive dentistry.

## 5 Conclusion

Within the limitations of this *in-vitro* study, sodium hypochlorite adversely affected resin-dentin bonding, with a more pronounced reduction observed in self-etch adhesive systems. Antioxidant pretreatment effectively reversed this impairment, with sodium ascorbate and nanocurcumin demonstrating superior and comparable improvement in shear bond strength across both adhesive strategies. These findings suggest that antioxidant application is a valuable adjunct when bonding to NaOCl-treated dentin, particularly for self-etch systems.

## Data Availability

The original contributions presented in the study are included in the article/Supplementary Material, further inquiries can be directed to the corresponding author.
